# Exploring Goal Attainment Scaling Among Occupational Therapy Students: A Retrospective Descriptive Analysis

**DOI:** 10.1155/oti/6034855

**Published:** 2025-01-15

**Authors:** Wafaa Alduraidi, E. Zhang, Lauren Foster, Lisa Mische-Lawson

**Affiliations:** Department of Occupational Therapy Education, University of Kansas Medical Center, Kansas City, Kansas, USA

**Keywords:** client-centered goal setting, goal attainment scaling, goal writing, occupational therapy education, professional training, student assessment, student competency

## Abstract

**Purpose:** This study explored the administration of goal attainment scaling (GAS) by entry-level occupational therapy (OT) students, examining their competency following minimal training. While GAS is valuable for measuring progress toward individualized client goals, its implementation requires specific skills and training that may challenge entry-level practitioners.

**Method:** Using retrospective data from a study of sensory garments' effects on children with autism spectrum disorder (ASD), we analyzed GAS administration by five entry-level OT students. Students received both indirect training through their curriculum and 3 h of direct research-specific preparation. Using a modified version of the GAS checklist, we evaluated students' competency through video recordings of GAS administration sessions and written GAS.

**Results:** Analysis revealed variable competency levels among students. While overall performance met 82.4% of checklist criteria, individual student competency varied considerably (54.5%–95.4%). Three of five students achieved the established 75% competency threshold. Students consistently met criteria for conceptual goal construction and timeframe specification but struggled with maintaining single dimensions of change and specifying observable behaviors.

**Conclusions:** Entry-level OT students demonstrated varying levels of competency in GAS administration following minimal training. Our findings suggest the need for more comprehensive training in specific aspects of GAS, particularly in scale construction and behavioral specification. These results have important implications for how OT programs prepare students to implement GAS in clinical practice.

## 1. Introduction

Goal attainment scaling (GAS) is an instrument for writing personalized evaluation scales to quantify progress toward defined goals after a period of intervention. The GAS process encompasses both the methodology of establishing goals and the measurement of outcomes. This process produces an individualized, criterion-referenced measure of a client's goal achievement that can be tracked and scored in a standardized manner to allow statistical analysis [1, 2]. The GAS scoring system uses a 5-point ordinal scale ranging from −2 to +2, with 0 representing *the expected level of goal attainment after intervention*. A score of +1 or +2 is provided *if a client accomplishes more than was expected based on preidentified criteria*; *if a client's achievement is less than expected*, also based on preidentified criteria, a score of −1 or −2 is given [3, 4] (see Table 1). The GAS process consists of two interconnected components: (1) the methodology of collaborative goal setting, which emphasizes establishing goals and levels of improvement meaningful to the client, and (2) the outcome measure, which assesses the efficacy of an intervention based on individually relevant goals [5]. This approach allows for the design of intervention outcomes specifically tailored for parent/caregiver, aligning with the client-centered occupational therapy (OT) philosophy [6].

The implementation of the GAS process follows three systematic steps: (1) determining individualized goals through a comprehensive parent/caregiver interview, (2) creating a scaling rubric to identify potential outcomes for each goal, and (3) scoring goal attainment at a later point [7]. Quality GAS goals must meet five criteria, or specific, measurable, attainable, relevant, and time-specific (SMART) [4]. These goals are established collaboratively between professionals familiar with the clients (such as clinicians or care providers) and the parents/caregivers before intervention begins [5, 7, 8]. This cooperation ensures everyone has a voice regarding what can be attained and agrees that the goal is worth pursuing [2]. Evidence suggests that collaborative goal setting increases the likelihood of goal achievement [2]. For each self-defined goal, the clinicians write expressive descriptions of five observable performance levels, established before intervention, making it potentially responsive to slight changes observed by parents/caregivers and clinicians as critical for daily functioning [9, 10]. These small changes are often not reflected in standardized tools, highlighting the unique value of GAS.

The success of the GAS process depends on two factors: the client's ability to work toward their goals and the clinician's expertise in anticipating realistic outcomes [2]. Literature reveals inconsistency regarding the level of training needed to competently administer the GAS. Some studies suggest that no specific training protocol is needed [11], while others recommend minimal training of 2-h sessions to introduce the GAS process, followed by practicing scale development with intensive feedback [4]. More comprehensive training of at least 12 h (2 h for general orientation, 5 h to develop goal-setting skills, and 5 h to improve goal-rating skills) has been recommended by other studies for the successful use of GAS [10, 12].

Kiresuk and Sherman first created GAS in 1968 for adults in community mental health programs [9]. Since then, it has undergone modifications and been applied in various rehabilitation disciplines [2, 13–17], psychiatry and mental health [7, 14–16], and pain management therapy [2, 14]. GAS has also been utilized with many clinical populations, including pediatric populations with developmental disabilities [8], sensory integration disorders [6, 16], autism [7, 18], and cerebral palsy [8, 11, 19, 20] as well as geriatric populations with multiple chronic conditions and functional limitations [3], psychiatric disorders [21], and cognitive disorders [3, 21]. GAS has unique applications as an applied clinical and research tool, especially when outcomes are unpredictable and there are no suitable standardized measures. Many traditional assessment tools are not designed to capture small, individualized changes or to measure outcomes across diverse interventions. GAS addresses these limitations by capturing the individuality of meaningful and relevant changes in occupational performance. It holds promising potential for OT for several reasons: It can detect subtle changes that are often missed by standardized instruments, it can be tailored to highly specific client goals, and it enables clinicians to assess intervention effectiveness in a personalized manner. Some evidence suggests it can be administered in a time-effective manner, further enhancing its utility in clinical settings [22]. Despite increasing research to support GAS efficacy and its use in various disciplines, little is known about how GAS is used in OT practice [23].

Due to its sensitivity to detect minor changes in performance, GAS has been rapidly expanding and applied in both clinical practice and research settings [24]. According to Grant and Ponsford [14], several benefits have been shown when GAS is used to assess the achievement of client-centered goals, including (1) assessing achievement using standardized, objective, and quantifiably identified levels of expected achievement, established before the beginning of the intervention and personally relevant to the client; (2) enabling evaluation of the effectiveness of an intervention across multiple levels and within and across individuals; (3) permitting comparisons of goal attainment across clients whose goals and interventions may differ significantly; and (4) enabling clients and their families to participate in the formulation of both the goals and the rubric for measuring goal achievement. They also stated that the use of GAS was associated with several therapeutic advantages, such as facilitating client participation in goal establishment, strengthening client–therapist rapport, promoting client motivation, and offering an easy method for evaluating performance and providing feedback to clients. GAS' ability to weigh goals according to clients' perceived priorities was another benefit.

Though there are many benefits to using the GAS to measure progress toward individualized goals, the implementation of GAS in clinical settings can be challenging, specifically related to the training and expertise required for clinicians to be reliable goal setters and raters. While some evidence suggests that the GAS is time-efficient to administer, other evidence shows that the GAS process is time-consuming in terms of conducting participant interviews, setting scaled goals, and performing follow-up goal ratings. To assure that bias is not present, goal setting and ratings must be regularly monitored [16]. It was shown that technical errors made during scale identification might significantly impact GAS' ability to evaluate goals [14]. Additionally, research has indicated that in order to obtain high reliability in GAS administration, clinical expertise (e.g., 1 year of full-time clinical experience) and training processes (e.g., approximately 12 h of training) may be necessary [10, 14]. The possibility that the GAS approach heavily depends on the ability of the clinician implementing it to provide valid, reliable, and meaningful scales is a critical drawback. Additionally, the GAS has been considered by some evidence to be more a measurement of how well a clinician can predict an outcome than an outcome measure itself [5].

## 2. Methods

### 2.1. Purpose

GAS has emerged as a widely utilized instrument among occupational therapists (OTRs) for measuring progress toward client-selected goals in both clinical practice and research settings. Evidence suggests that effective GAS administration requires adherence to specific process to ensure validity, reliability, and meaningfulness of outcomes; however, there is limited research examining this process, particularly among entry-level practitioners. The purpose of this study is to explore the administration of the GAS by entry-level OT students. Through systematic review of literature on clinical competency assessments and consultation with experienced OT educators, we established a benchmark for competent GAS administration. Literature indicates that clinical competency assessments in health professional education typically employ a threshold of 70%–75% as minimum passing scores [25, 26]. Building on this established standard, we determined that a 75% achievement rate on a modified GAS checklist would represent an appropriate competency threshold for entry-level practitioners. This benchmark strategically balances the imperative for maintaining high-quality care standards while acknowledging the developmental nature of clinical skills among entry-level practitioners.

Therefore, we hypothesize that entry-level OT students will demonstrate competent GAS administration by meeting or exceeding 75% of the modified GAS checklist criteria following minimal training. Testing this hypothesis will provide critical insights into whether brief training protocols can produce satisfactory levels of competence in GAS administration or if more intensive training approaches might be necessary for entry-level practitioners.

### 2.2. Design

This study is an exploratory descriptive analysis of retrospective data from a larger study investigating the effects of sensory garments on 19 children with autism spectrum disorder (ASD) [27]. This is an appropriate research design for utilizing existing data sets to document the behaviors of individuals or groups, represent attribute variables, explain how variables change over time using quantitative methods, and/or systematically reinvestigate relationships between two or more variables [28].

### 2.3. Participants/Setting

In the original study by Mische Lawson et al. [27], GAS was administered virtually via secure videoconferencing software (Zoom) to identify and measure progress toward goals. These virtual sessions typically included the OT student, the parent(s) of the child participant, and occasionally the child, depending on age and ability to participate. No other researchers were present during these sessions to maintain a naturalistic environment. The study employed a pretest–posttest repeated measures design to evaluate the effectiveness of sensory garments. Assessments were conducted at three timepoints: baseline, 4 weeks, and 8 weeks of garment use, allowing for systematic tracking of changes in child participation, parental competence, and parent stress.

For this retrospective analysis, participants were the five entry-level OT students on the research team who administered the GAS. The OT student participants received training that occurred in two phases: indirect classroom instruction and direct research-specific preparation. The indirect training, as part of their entry-level OT curriculum, included comprehensive instruction on writing SMART goals through interactive lecture and practical activity where students participated in a group exercise analyzing written goals to identify SMART versus non-SMART goals, enhancing their understanding of goal-writing criteria. Following this, students received orientation to GAS principles and its clinical applications as an assessment tool. This classroom preparation culminated in a practical activity where students wrote GAS for goals identified during their clinical placements. Several months after completing this classroom training, students participated in the original study, where they received direct research-specific virtual training via Zoom platform. This training included (1) a 1-h overview by the principal investigator (PI) introducing GAS use in the research context; (2) a 1-h virtual training session by a licensed OTR with over 10 years of clinical experience, covering GAS administration, interviewing techniques, and study procedures; and (3) 1 h of individual observation where each student watched an experienced OTR conduct an initial GAS session with a parent–child dyad. Following this 3-h direct training, students independently administered GAS during initial meetings with 14 parent participants. While weekly 1-h virtual research meetings were available throughout the 14-week study period for general questions and support, these were not considered part of the formal training hours. This training approach provided essential preparation for the study's GAS administration within the constraints of the research timeline and resources.

### 2.4. Materials/Instrumentation

In the original study, researchers employed two primary assessment tools: (1) Canadian Occupational Performance Measure (COPM): This client-centered outcome measure was chosen for its ability to identify areas of importance, satisfaction, and performance from the parents' perspective. The COPM provided a structured framework for initiating discussions about the child's daily routines and prioritizing areas for intervention; (2) GAS: It was selected for its capacity to capture individualized progress in heterogeneous populations, making it particularly suitable for children with autism where standardized measures might not adequately reflect subtle but meaningful changes. GAS allowed for the creation of personalized, scaled goals based on the priorities identified through the COPM. The combination of COPM and GAS allowed for a transition from broad areas of concern to specific, measurable goals tailored to each child's unique needs. The process of creating GAS goals was guided by a semistructured interview protocol, designed to bridge the insights gained from the COPM with the specific requirements of GAS. This protocol facilitated a collaborative dialogue between OT students and parents, ensuring that goals met SMART criteria and were appropriately scaled on the 5-point GAS ordinal scale.

For this retrospective analysis, which focuses solely on the GAS administration process, two instruments were used: (1) GAS for setting and measuring progress toward the identified goals and (2) a GAS checklist to examine if entry-level OT students administered the GAS competently.

#### 2.4.1. GAS

The original study utilized a modified version of GAS. While traditional GAS typically uses a 5-point ordinal scale (−2 to +2) with 0 representing the *expected outcome* [10], the original study employed a scale where 0 represented *baseline performance*. This modification, though different from traditional scoring, aligns with evolving practices in GAS implementation. A recent scoping review by Logan et al. [29] highlighted considerable variability in GAS scoring and implementation across different studies and disciplines, noting that while the original scaling used 0 as the *expected outcome*, some researchers have adopted alternative approaches, including using 0 as *baseline*, to address criticisms that baseline performance is not clearly indicated in the standard scale. This modified GAS format was particularly relevant for the study's context, where it was used to measure progress in children with ASD, a population where individualized outcome measurement is crucial due to the heterogeneous nature of intervention responses. GAS' value in this context lies in its ability to capture individualized progress and subtle changes that might not be reflected in standardized measures. This is particularly important when working with children with sensory integration disorders, where outcomes are commonly diverse and highly individualized [6]. Research has demonstrated preliminary reliability evidence supporting GAS as a meaningful outcome measure for children with ASD [16].

In this retrospective analysis, we examined the written GAS from the original study, where scores of +1 and +2 indicated improvement beyond baseline, reflecting progressive levels of achievement. Our focus was on analyzing how entry-level OT students implemented the process of GAS, including their ability to create clear, measurable levels and maintain consistent scaling across goals. As this is a retrospective analysis, we maintained the original study's scaling method, examining the technical aspects of how students constructed and documented the scales rather than focusing on the scaling approach.

#### 2.4.2. GAS Checklist

For this study, we adapted the GAS checklist from King et al.'s [20] original version. Our modified checklist contains components that could be directly observed from video recordings of GAS administration sessions and written GAS, aligning with our retrospective analysis methodology. The modified checklist consisted of 11 criteria across three main sections: (1) the process of selecting goal areas: (a) goals are meaningful and relevant to each others and (b) goals make sense from a conceptual point of view; (2) the scale as a whole should (a) have levels that reflect clinically meaningful gradations of improvement; (b) have approximately equal intervals between the goal attainment levels; (c) specify a time period for achievement; (d) reflect a single dimension of change, keeping other variables constant; and (e) not reflect attainment that is dependent on the therapist's physical assistance (unless explicitly stated in the goal); (3) each of the levels on the scale should (a) be written as clearly as possible, in concrete behavioral terms; (b) specify an observable behavior of the client; (c) be written in the present tense; and (d) be achievable or realistically possible.

Assessment of each criterion was based on King et al.'s [20] framework for ensuring the technical quality of the GAS procedure, with a key distinction in our data sources. The first section, focusing on the process of selecting goal areas, was assessed from the video recordings of the virtual interviews. Goal meaningfulness and relevance were evaluated by observing the goal-setting process and verifying that selected goals corresponded to areas rated as high priority (score of 10) on the COPM, aligning with King et al.'s [20] recommendation for collaborative goal setting. The second and third sections of criteria were assessed from the written GAS for each child participant from the original study. This allowed for a detailed examination of the technical aspects of scale construction and level descriptions. We evaluated whether the scales showed clear, meaningful gradations; had approximately equal intervals; specified a time frame; focused on a single dimension of change; and appropriately considered therapist assistance. For individual-scale levels, we assessed clarity, use of concrete behavioral terms, specification of observable behaviors, use of present tense, and realistic achievability.

Criteria were marked as met if observed in either the video recordings (at any point) or written scales, as appropriate. One point was assigned per met criterion, allowing for a maximum score of 11. Our adapted checklist necessarily excluded certain aspects of GAS administration that could not be directly observed from our available data, including the administrator's clinical experience, the process of insuring adequate goal rating by independent raters, and the methodology of determining the summary scores. This checklist served to review the goal-setting and scaling procedures of entry-level OT students, exploring their competence in GAS administration.

### 2.5. Procedures

The original study followed a structured protocol for GAS administration. After completing their training, entry-level OT students conducted initial interviews with parents of children with ASD using a three-part format. First, students administered the COPM to identify priority areas and assess current performance levels. Second, they engaged in GAS goal setting and scale creation, translating COPM priorities into individualized GAS goals. During this process, students guided parents to identify goals that could be improved by wearing the selected sensory garments. Parents prioritized these goal areas using the COPM, with areas scoring 10 indicating the highest priority and meaningfulness. These prioritized areas were then developed into GAS. The original study utilized a modified 5-point ordinal scale where 0 represented *the child's baseline performance*, +1 and +2 indicated im*provements beyond baseline after wearing the sensory garment*, and −1 and −2 represented *performance worse than baseline following intervention*. The final part of parents' interviews addressed study logistics and sensory garment selection. Students independently administered GAS with 14 parent participants (approximately three interviews administered by each student), with all sessions recorded. Goal performance was assessed at three follow-up timepoints (baseline, 4 weeks, and 8 weeks) across the 14-week study period to track intervention effects of sensory garments.

For this retrospective analysis, we conducted a systematic examination of two data sources from the original study: video recordings of GAS administration sessions and written GAS. Prior to beginning the analysis, both authors (W.A. and L.M.-L.) independently reviewed King et al.'s [20] recommendations for effective GAS implementation (see Table 2) and the table of common errors in writing GAS to ensure understanding of GAS checklist criteria (see Table 3). This structured approach helped ensure consistent evaluation of student-written GAS across all cases. The usability of the modified GAS checklist was confirmed by both authors independently rating two randomly selected GAS and their corresponding interview recordings, establishing > 80% interrater reliability. This process confirmed that the modified GAS checklist was a suitable tool for analyzing the data.

Our observation process evaluated two distinct components of GAS administration competency. First, we examined video recordings of the initial interviews to assess students' proficiency in conducting the goal-setting process. Second, we analyzed the written GAS to evaluate their technical construction and documentation. The first author (W.A.) logged the duration, in minutes, of the GAS process in each video recording and reviewed all video recordings and written GAS using the modified GAS checklist.

Each video recording was examined in its entirety, focusing particularly on the GAS goal-setting portion. Written scales were evaluated for technical components including scale construction, level descriptions, and documentation format. Each criterion on the modified checklist was marked as met or unmet based on observable evidence from either the video recordings or written scales, as appropriate to the specific criterion. A second rater (L.M.-L.) independently completed the checklist for 20% of the videos to ensure consistency in the evaluation process.

>The university's institutional review board approved the study, Protocol #00146392, on October 8, 2020. Informed consent was obtained from all parent participants in Lisa Mische-Lawson's original study in conformity with the Declaration of Helsinki. The retrospective analysis adhered to the guidelines set forth by the Declaration of Helsinki. KUMC Institutional Review Board determined that informed consent was not required for the retrospective analysis of GAS administration by the study team.

### 2.6. Competency Assessment

Competency was evaluated at two distinct levels. First, individual student performance was assessed using a 75% threshold, whereby students needed to correctly demonstrate 75% or more of the required competency criteria to be considered competent. In addition to the individual student performance, we also conducted a higher-level analysis of how often each individual competency criterion was met across all student administrations. This criterion-level analysis provided insight into which specific competencies were most frequently demonstrated, versus those that proved more challenging.

### 2.7. Analysis

Researchers used the Statistical Package for Social Sciences (SPSS) 27 to calculate descriptive statistics (e.g., frequencies, mean, range, and percentages), summarize participant characteristics, and assess OT student performance on the modified GAS checklist, including subscales and total scores. Competency was defined as meeting 75% of the total checklist criteria. Examples of GAS meeting and not meeting criteria are presented qualitatively.

## 3. Results

The participants in this study were five entry-level OT students (all female) in their mid-20s enrolled in the Master of Science in Occupational Therapy program at the KUMC. Students received both indirect training through their OT curriculum (classroom instruction on GAS principles and SMART goal writing) and 3 h of direct virtual training specifically for this research. None had prior experience administering GAS in clinical settings. Each student conducted approximately three interviews with parents of children with ASD.

The data analyzed included 14 initial interviews from the original study of 19 parent interviews. Five interviews were excluded: four conducted by the licensed OTRin the research team and one with missing video recording. The 14 interviews were conducted with parents of children with ASD (13 male, one female; ages 4–13 years, mean = 8.2 years). Prior to rating all videos, the usability of the GAS checklist was confirmed. A second rater (L.M.-L.) completed the GAS checklist with 20% of the videos, and a rater reliability of 84.8% was confirmed (82%–91%) with the first author (W.A.); no modifications to the GAS checklist were needed.

Across all 14 GAS administrations, students consistently met two criteria: Goals made sense from a conceptual point of view (Criterion 1.2) and scales specified a time period for achievement (Criterion 2.3). The lowest performance was observed in Criterion 2.4 (the scale should reflect a single dimension of change), met in only 50% of administrations. Criterion 3.2 (each level of the scale should specify an observable behavior of the client) was met in 64.2% of cases (see Table 4). Analysis of student performance using the modified GAS checklist revealed that 82.4% of the checklist criteria were met overall. However, individual student performance varied considerably. Three of the five students met the established competency threshold of 75% of checklist criteria, while two did not achieve this benchmark. Student 1 met 54.5% and Student 2 met 72.7% of the criteria (see Table 5).

Table 6 provides examples of GAS meeting and not meeting checklist criteria, illustrating common patterns in student performance including the use of multiple dimensions of change and overly generalized behaviors in goal writing.

## 4. Discussion

The results did not support our hypothesis that entry-level OT students could administer GAS competently with minimal training. Individual analysis using the 75% threshold on the modified GAS checklist revealed varying levels of competency: Three students demonstrated competent administration (achieving > 75% of criteria), while two did not meet this benchmark. Performance varied considerably, ranging from 54.5% to 95.4% of checklist criteria met. Several factors may explain this variability in GAS administration competency. The literature emphasizes that effective GAS administration requires substantial clinical experience, with scholars recommending at least 1 year of clinical practice to develop necessary skills in goal setting and outcome prediction [10]. Our entry-level students had not yet developed this clinical experience depth, potentially affecting their ability to translate client priorities into structured GAS goals.

Our examination of video recordings revealed specific technical challenges in GAS administration. Only half of the GAS reflected a single dimension of change (Criterion 2.4), and 64.2% specified observable client behaviors (Criterion 3.2). These technical difficulties manifested in two ways: scales that combined multiple aspects of performance and behavioral descriptions lacking specificity. These findings align with research indicating that developing technical proficiency in GAS requires extensive training and practice [10, 14]. Through systematic analysis of the video recordings, we observed that students who did not meet the competency threshold often transcribed parents' statements verbatim rather than translating them into measurable GAS goals. This approach reflected beginning-level clinical reasoning skills, where practitioners may struggle to synthesize and transform client input into structured, measurable goals [30]. This observation aligns with broader evidence that many healthcare practitioners, not only entry-level OT , face challenges in converting client priorities into observable, objective measures [5, 31].

Students demonstrated consistent success in two areas: conceptual goal construction (Criterion 1.2) and timeframe specification (Criterion 2.3). This consistency reflects the structured protocol of the original study, which provided clear parameters for goal timeframes and utilized the COPM framework. While the COPM offered a systematic approach to identifying goal areas, the skills required for effective GAS administration differ significantly. The process of translating COPM-identified priorities into properly scaled GAS goals requires distinct interviewing and analytical skills that may require specific training beyond COPM familiarity.

Based on the findings, we propose several strategies to enhance GAS administration training for entry-level OT students. First, expanded technical training should focus specifically on writing goals with single dimensions of change, specifying observable behaviors, and translating client priorities into measurable goals. Second, implementing structured practice opportunities, including case scenario discussions, peer review sessions, and regular feedback from experienced practitioners, could support skill development [4, 16, 18, 23]. Training programs should incorporate repeated practice opportunities, as research indicates that technical proficiency in goal writing improves with experience [10]. While our data suggested some improvement in student performance across multiple administrations, this observation was based on video analysis rather than systematic measurement of improvement over time. These recommendations arise directly from our analysis of student performance patterns and align with literature suggesting that comprehensive training enhances GAS administration competency [10, 14, 18]. The specific challenges identified in our analysis—particularly in maintaining single dimensions of change and specifying observable behaviors—provide clear direction for focusing training efforts.

## 5. Limitations and Future Directions

The use of retrospective data was both a strength and a limitation of this study. While retrospective nature of our analysis allowed for a comprehensive review of student performance, it may limit our ability to fully understand the students' experiences with GAS administration. We could not question students about specific challenges they encountered or their reasoning behind certain goal-writing decisions. Additionally, we could not validate with parents whether the established goals truly reflected their priorities and concerns.

Our training approach had several limitations. First, while students received training in both COPM and GAS, these assessment tools require distinct interviewing and goal-setting skills. The differences between COPM and GAS interviewing techniques were not explicitly addressed in the training, potentially affecting students' ability to effectively translate COPM-identified priorities into GAS goals. Second, the nonstandard approach of conducting three follow-up assessments, rather than the typical single postintervention assessment, may have influenced how students constructed their GAS.

The small sample size of five students and 14 interviews limits the generalizability of the findings. While this allowed for detailed analysis of individual performance, it may not represent the broader population of entry-level OT students. Additionally, all students were from the same educational program, potentially limiting the diversity of educational experiences represented. Technical limitations included our inability to assess certain aspects of GAS administration that could not be directly observed from video recordings, such as students' clinical reasoning processes or their understanding of goal scaling. The modified checklist, while appropriate for our retrospective analysis, excluded some components of King et al.'s [20] original checklist that might have provided additional insights into student competency.

Future research should explore the effects of different instructional approaches for developing OT student competency in administering GAS, particularly for students requiring greater support to develop competency. Future research should also investigate differences between how new and experienced OTs administer GAS. Furthermore, future research could explore the integration of the GAS administration process into the OT curriculum, including the incorporation of practical opportunities for students to practice administering the GAS under supervision and receiving feedback. This could help to ensure that students are adequately prepared for the challenges of administering the GAS in real-world practice settings.

## 6. Implications for OT Practice

Based on our findings, we propose three key implications for OT practice:

• Entry-level OT education must expand beyond basic GAS principles to include comprehensive training in goal setting, scaling, and interviewing techniques.
• Clinical settings should establish structured mentorship and supervision protocols for new graduates implementing GAS, ensuring competency before independent administration.
• Professional development opportunities should focus specifically on bridging identified gaps between academic preparation and clinical application of GAS.

## 7. Conclusion

GAS is a valuable tool in OT practice for client-centered goal setting and individualized progress measurement. This study demonstrated that entry-level OT students showed variable competency in GAS administration with minimal training, with three of five students meeting established competency criteria. These findings highlight the complexity of GAS administration and the need for more comprehensive training approaches. Our results emphasize the importance of structured preparation for entry-level practitioners using GAS. OT education programs should consider implementing more comprehensive training protocols that address both technical aspects of GAS and the clinical reasoning skills needed for effective implementation. This includes focused instruction on maintaining single dimensions of change, specifying observable behaviors, and translating client priorities into measurable goals.

These findings have direct implications for how OT programs prepare students to use GAS in practice. Structured training, ongoing supervision, and multiple opportunities for practical application appear essential for developing competency in GAS administration. By acknowledging these needs early in professional education, we can better prepare entry-level practitioners to effectively utilize this important clinical tool.

## Figures and Tables

**Table 1:** The GAS 5-point ordinal scale.

**Scale**	**Predicted attainment**
−2	*Much less than expected*
−1	*Less than expected*
0	*Expected outcome of goal attainment*
+1	*Greater than expected*
+2	*Much greater than expected*

**Table 2:** Recommendations for effective goal attainment scaling implementation.

**Questions to consider**	**Criteria to be met**	**Procedures and tools to help ensure criteria are met**
1. How can one ensure adequate goal selection?	The process of selecting goal areas should ensure that 1. Goals are meaningful and relevant to others 2. Goals make sense from a conceptual point of view (e.g., if the goals of the program are to improve day-to-day function, then the majority of goals set should be functional in nature, rather than impairment based)	Employ collaborative goal setting (therapists select broad goal areas in conjunction with knowledgeable others such as the teacher, parent, and/or child) Use peer review of goal content

2. How can one ensure adequate goal scaling (i.e., adequately written goal levels on the 5-point scale)?	Each of the levels on the scale should 1. Be written as clearly as possible, in concrete behavioral terms 2. Specify an observable behavior of the child 3. Be written in the present tense 4. Be achievable or realistically possible The scale as a whole should 5. Have levels that reflect clinically meaningful gradations of improvement 6. Have approximately equal intervals between the goal attainment levels (i.e., the change from +1 to +2 should be similar to those between ∗2 and ∗1) 7. Specify a time period for achievement 8. Reflect a single dimension of change (as long as a goal remains meaningful), keeping other variables constant 9. Not reflect attainment that is dependent on the therapist's physical assistance (unless the assistance of others is a written part of the goal)	Each criterion can be assured through the use of three interconnected procedures and tools: 1. Therapist training 2. Peer review to ensure the adequacy of the goal scales 3. Use of a standard procedure and checklist to review the technical adequacy of written goal scales

*Note:* Adapted from King et al. [20]. These guidelines and criteria are based on the authors' clinical experience with GAS implementation and synthesis of recommendations from the literature.

**Table 3:** Common errors in creating GAS.

**Error**	**Description**	**Solution**
Overly generalized goals	If the expected level (i.e., 0 level) of a scale is written in very general terms (e.g., “walks a greater distance in a set period with assistance”), it will be difficult or impossible to create the remaining scale points, therefore making the goal unmeasurable	The expected level of a scale should be written as clearly as possible (e.g., “walks with platform walker 100 m in 6 min with two hands on the walker to assist with steering”)
Overly technical goals	A goal setter may use terms specific to his/her profession in creating a scale that the goal rater is not familiar with	Write goals in common terms, especially if the goal rater is not of the same professional background as the goal setter
Multiple variables of change	A scale may include two or more variables of change. This could be problematic if the scale is written so that change is expected to occur simultaneously on these variables	Decide on one variable by which to measure change in performance and hold others constant. If in doing so, the goal does not remain meaningful, two variables could change within in a single scale, provided that each scale level differs on only one variable
Unequal scale intervals	A scale may be created where the amount of clinical change is greater between, say, the +1 and +2 levels than the amount of change between the −2 and −1 levels	Aim for clinically equal intervals between all levels of the scale
Clinically irrelevant or unrealistic scale levels	A scale may be created where one or more of the levels represents an amount of change that would not be clinically relevant to the child (i.e., the amount of change is too small to matter) or the amount of change is unrealistic for the child (i.e., the amount of change is too great)	The amount of change between all scale levels needs to be clinically relevant, and all levels should be achievable for the child
Using different tenses (i.e., past, present, and future) when writing scale levels	A GAS may be written with the −2 level written in one tense and all other levels in another tense, which could be confusing and bias the goal rater	All scale levels should be phrased in the present tense, in order for evaluation to make sense at different time points (i.e., “walks …”)
Redundant or incomplete scale levels	A scale may be written where a child could be scored on two levels at the same time (e.g., the +1 level has *walking distances specified between “40 and 50 m”* and the +2 level specifies *distances between “50 and 60 m”*). If a child walks exactly 50 m, both the +1 and the +2 levels would be correct. On the other hand, a gap could be present in the scale where a child could not be scored on any level (e.g., the +1 specifies *walking distances between “40 and 50 m”* and the +2 specifies *distance between “60 and 70 m”*; if a child walks 55 m, neither the +1 nor the +2 level is correct)	Be careful not to create scale levels that are redundant or incomplete. Careful wording (e.g., +1 would be *“more than 40 m and up to 50 m”* and +2 would be *“more than 50 m and up to 60 m”*) or specific instructions to the rater (e.g., if a child obtains a midway point between two levels, score the child at the lower level) will be of benefit
Blank scale levels	It may be difficult to write the more extreme levels of a scale, tempting the goal setter to leave these levels blank. If a child happens to achieve an upper or lower extreme, it would be impossible to rate the child's performance	Be careful to set goals where it is possible to complete all scale levels

*Note:* Adapted from King et al. [20].

**Table 4:** The overall performance on each criterion of GAS checklist.

**Criteria**	**Number of met criteria**	**Percentage (%)**
1. The process of selecting goal areas		
a. Goals are meaningful and relevant to others	13	92.9%
b. Goals make sense from a conceptual point of view (e.g., if the goals of the program are to improve day-to-day function, then the majority of goals set should be functional in nature, rather than impairment based)	14	100%
Average for Section 1	13.5	96.4%
2. The scale as a whole should		
a. Have levels that reflect clinically meaningful gradations of improvement	12	85.7%
b. Have approximately equal intervals between the goal attainment levels (i.e., the change from +1 to +2 should be similar to that between −2 and −1)	10	71.4%
c. Specify a time period for achievement	14	100%
d. Reflect a single dimension of change (as long as a goal remains meaningful), keeping other variables constant	7	50%
e. Not reflect attainment that is dependent on the therapist's physical assistance (unless the assistance of others is a written part of the goal)	11	78.6%
Average for Section 2	10.8	77.1%
3. Each of the levels on the scale should		
a. Be written as clearly as possible, in concrete behavioral terms	12	85.7%
b. Specify an observable behavior of the client	9	64.2%
c. Be written in the present tense	13	92.9%
d. Be achievable or realistically possible	12	85.7%
Average for Section 3	11.3	82.1%
Average total criteria met	11.5	82.4%

**Table 5:** Variability of student administration of goal attainment scaling.

**GAS video ID**	**Duration of GAS administration process in minutes**	**Total number of criteria met**	**% criteria met**
Student 1			
14	13	4	36.3%
21	9	8	72.7%
Average	11	6	54.5%
Student 2			
7	22	5	45.4%
15	16	11	100%
22	6	8	72.7%
Average	14.6	8	72.7%
Student 3			
11	7	10	90.9%
17	7	10	90.9%
24	5	10	90.9%
Average	6.3	10	90.9%
Student 4			
8	6	11	100%
16	4	10	90.9%
Average	5	10.5	95.4%
Student 5			
6	17	11	100%
12	5	9	81.8%
18	6	9	81.8%
25	8	11	100%
Average	9	10	90.9%
Average for all students	9.18	9	82.4%

**Table 6:** Examples of GAS meeting and not meeting checklist criteria.

**−2**	**−1**	**0**	**+1**	**+2**
GAS example meeting all checklist criteria				
After taking a shower and donning garments, child takes 45 min or more to calm and be ready for bed	After taking a shower and donning garments, child takes 35 min to calm and be ready for bed	After taking a shower and donning garments, child takes 25 min to calm and be ready for bed	After taking a shower and donning garments, child will be calm and ready for bed in 15 min	After taking a shower and donning garments, child will be calm and ready for bed in 5 min or less
GAS example not meeting checklist Criteria 2.1, 2.2, 2.4, 3.1, 3.2, and 3.4				
Says “bye reading,” sits and waits	Does not open book, whines	When instructed to read, child turns pages quickly, does not pronounce every word (“swallows words”), does not show understanding of what he read	Turns pages slower, reads all the words but does not pronounce every word	Reads and pronounces all the words, shows understanding of what he read
GAS example not meeting checklist Criteria 2.1, 2.2, 2.4, 2.5, 3.1, 3.2, and 3.4				
Child would not put head/arms through shirt	Child would take a longer period of time to complete dressing	Child mother grabs and orients for all clothing items	Child would complete 50% of dressing routine independently	Child would independently dress self

## Data Availability

The data that support the findings of this study are available from Dr. Lisa Mische-Lawson, but restrictions apply to the availability of these data, which were used with permission for the current study and so are not publicly available. Data are however available from the authors upon reasonable request and with permission of Dr. Lisa Mische-Lawson.
